# Stem cells supporting other stem cells

**DOI:** 10.3389/fgene.2013.00257

**Published:** 2013-12-03

**Authors:** Judith Leatherman

**Affiliations:** School of Biological Sciences, University of Northern ColoradoGreeley, CO, USA

**Keywords:** stem cell niche, stem cell therapy, hair follicle stem cell, hematopoietic stem cells, *Drosophila* stem cells, germline stem cell, self-renewal pathways

## Abstract

Adult stem cell therapies are increasingly prevalent for the treatment of damaged or diseased tissues, but most of the improvements observed to date are attributed to the ability of stem cells to produce paracrine factors that have a trophic effect on existing tissue cells, improving their functional capacity. It is now clear that this ability to produce trophic factors is a normal and necessary function for some stem cell populations. *In vivo* adult stem cells are thought to self-renew due to local signals from the microenvironment where they live, the niche. Several niches have now been identified which harbor multiple stem cell populations. In three of these niches – the *Drosophila* testis, the bulge of the mammalian hair follicle, and the mammalian bone marrow – one type of stem cell has been found to produce factors that contribute to the maintenance of a second stem cell population in the shared niche. In this review, I will examine the architecture of these three niches and discuss the molecular signals involved. Together, these examples establish a new paradigm for stem cell behavior, that stem cells can promote the maintenance of other stem cells.

## INTRODUCTION

The field of stem cell biology has seen numerous studies over the years touting the benefits of stem cell therapies. Injection of various types of adult stem cells were able to improve conditions such as myocardial infarction, spinal cord injury, and muscle degeneration (McDonald et al., 1999; Orlic et al., 2001; Dezawa et al., 2005). In these early studies, it was originally assumed that the benefits arose from true tissue regeneration due to stem cell differentiation into specific cell types. However, further examination of these improvements revealed that very few stem cell-derived cells were actually incorporated long-term into the tissues of interest. It is now well appreciated that stem cells secrete paracrine factors which have a trophic, cell protective effect on extant tissue cells, and much of the improved tissue functionality in disease models can be attributed to this effect, rather than new cells from the stem cells (Zandonella, 2005; Caplan and Dennis, 2006; Gnecchi et al., 2008; Pelacho and Prosper, 2008; Uccelli et al., 2011).

Does this idea that stem cells secrete a “special juice” have anything to do with the normal functioning of stem cell populations? Recent findings in three different adult stem cell niches – the *Drosophila* testis, the mammalian hair follicle, and the mammalian bone marrow – provide evidence that it does. Each of these stem cell niches harbor two separate populations of stem cells, and in each example, one stem cell population has been found to provide important molecular signals that keeps the other self-renewing.

## THE *Drosophila* TESTIS NICHE

In the *Drosophila* testis, sperm production is maintained over the lifetime of adult flies by continual division of two stem cell populations, the germline stem cells (GSCs) and the cyst stem cells (CySCs). Why two stem cell populations? Just as in mammalian spermatogenesis, the germ cells must be guided through the differentiation process by specialized somatic cells; in mammals these are the Sertoli cells, and in *Drosophila* they are the cyst cells. Both Sertoli and cyst cells completely engulf germ cells within their cytoplasm, providing important differentiation cues. However, while Sertoli cells are long-lived cells that are re-used by each group of differentiating germ cells, *Drosophila* cyst cells associate with each packet of differentiating germ cells, do their job of germ cell guidance, and then die. Thus, new cyst cells must also be continually produced by a stem cell population, and sperm production is dependent on *both* stem cell populations. The two stem cell populations must also coordinate their division rates, since their differentiating progeny associate with each other, with two cyst cells for each differentiating germ cell.

**FIGURE 1:**
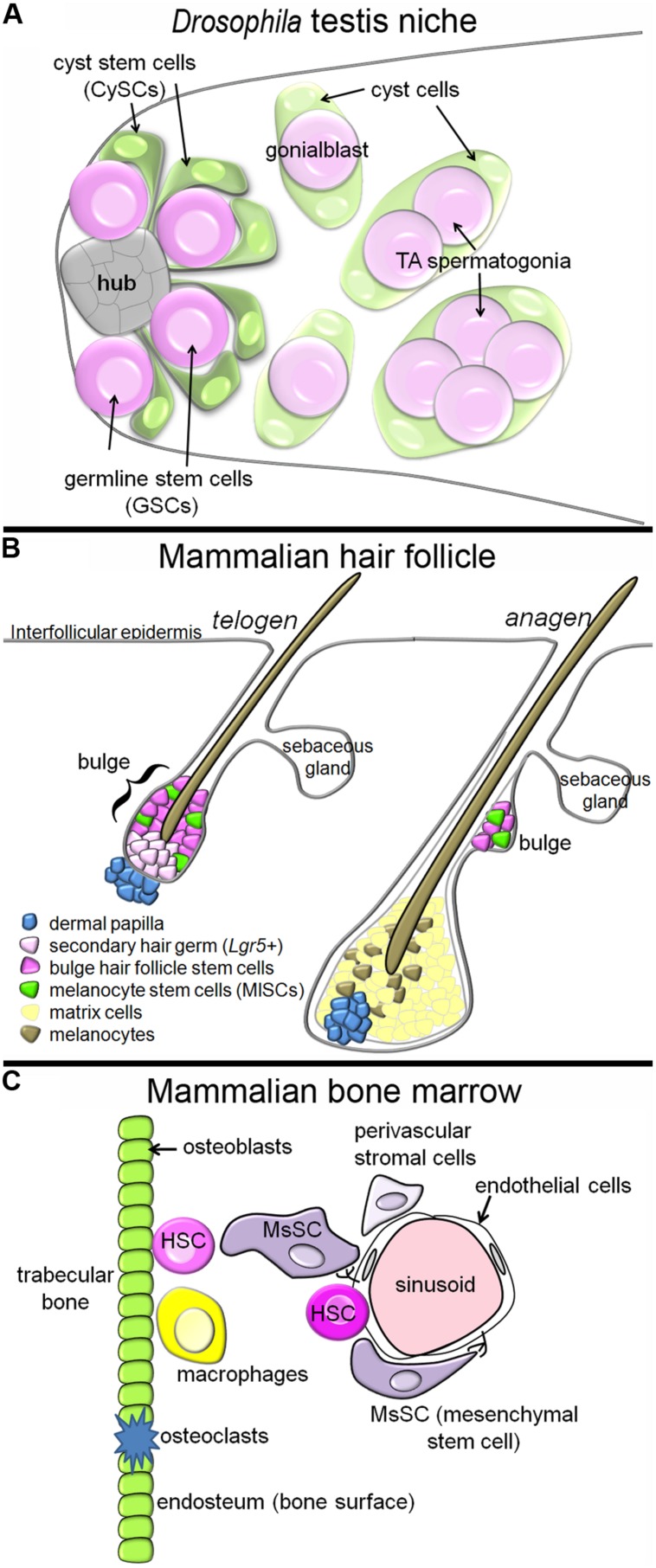
**Tissue architecture of three stem cell niches. (A)** In the *Drosophila* testis niche, two stem cell populations, the GSCs and CySCs, intermingle around a cluster of cells called the hub. When the stem cell populations divide, daughters that move away from the hub differentiate, and the differentiating germ cells, which begin to undergo TA, become encysted by the differentiating cyst cells. In this niche, the CySCs produce signals promoting the self-renewal of neighboring GSCs. **(B)** In the mammalian hair follicle, the bulge and sHG HFSCs reside next to the dermal papilla during telogen, and the MlSC intermingle with the HFSCs. During anagen, the HFSCs and MlSCs divide to produce matrix cells and melanocytes, respectively, which cluster around the dermal papilla and contribute to growth of the new hair. The HFSCs provide molecular signals a different stages of the hair follicle cycle which regulate the behavior of the MlSCs. **(C)** In the mammalian bone marrow, HSCs have been identified next to sinusoids (blood vessels) and next to the endosteum (osteoblasts). MsSCs, which are innervated by the sympathetic nervous system, cluster around the sinusoids, and are required for HSC maintenance. Other cell populations with reported contributions in this niche are the endothelial cells, macrophages, osteoclasts, and other perivascular stromal cells.

The two stem cell populations share a common niche, intermingling around a group of cells called the hub (Hardy et al., 1979). When either type of stem cell divides, the daughter cell that stays in contact with the hub self-renews, while the daughter that loses contact with the hub will differentiate (Yamashita et al., 2003). As soon as a differentiating germ cell moves away from the hub, it is called a gonialblast, and it is immediately engulfed, or encysted, by two cyst cells. As differentiation commences, the gonialblast undergoes a transit amplification (TA) period, followed by meiosis. The cyst cells, in contrast, immediately withdraw from the cell cycle upon exit from the niche, and simply stretch their cytoplasmic arms to engulf the growing group of differentiating germ cells (**Figure 1A**).

It was originally thought that the two stem cell populations in this niche self-renewed independently from each other, both in response to the secreted cytokine Upd from the hub, which activates Jak/STAT signaling in the stem cell populations. Ectopic Upd expression caused ectopic accumulation of both stem cell types through the whole testis, and STAT inactivation led to loss of both stem cell populations (Kiger et al., 2001; Tulina and Matunis, 2001). The first hint that the situation was a bit more complex came from studies of the Jak/STAT target gene in CySCs, *zfh-1* (Leatherman and Dinardo, 2008). Zfh-1 is normally restricted to CySCs, and when expression was artificially maintained in cyst cells, differentiation was prevented and excess stem-like cyst cells accumulated through the testes. Interestingly, accompanying these excess CySC-like cells were also ectopic GSCs, even though the germline cells had received no genetic manipulation themselves. Since this phenotype was similar to that observed with ectopic expression of the hub ligand Upd, the authors next activated Jak/STAT signaling cell autonomously in either the cyst lineage or germ lineage cells. They found that constitutive Jak/STAT in the germ lineage had no effect, while constitutive Jak/STAT in the cyst lineage caused ectopic accumulation of *both* stem cell populations. Thus, CySCs away from the hub are able to support ectopic GSCs, and this was the first reported example of one stem cell population supporting the self-renewal of a second stem cell population in a shared niche (Leatherman and Dinardo, 2008).

Subsequent studies of this niche have attempted to determine whether the CySCs are *necessary* for GSC self-renewal during normal tissue homeostasis. Since *stat* and *zfh-1* are required for CySCs to be maintained as stem cells, these genes were knocked down by RNAi to test whether loss of CySCs also led to GSC loss. Depletion of either gene product in cyst lineage cells caused a significant reduction in the number of CySCs, which in turn led to a significant reduction in the number of GSCs, further supporting the notion that CySCs are required for GSC maintenance (Leatherman and Dinardo, 2010; Issigonis and Matunis, 2012). Another study provided confusingly mixed results. In this study the apoptotic gene *grim* was transiently activated in cyst lineage cells, and this treatment led to ablation of all CySCs and cyst cells in about 80% of the testes. After 1 week, half of the testes had lost all germline cells, while the other half continued to maintain germline cells that failed to differentiate (Lim and Fuller, 2012). In summary, while it is clear that CySCs have the capability to support GSC self-renewal, it is still an open question whether CySCs are absolutely required for GSC maintenance in the setting of the normal niche.

What are the signals produced by CySCs that promote GSC maintenance? GSCs are known to require Jak/STAT and BMP signaling for their maintenance (Kiger et al., 2001; Tulina and Matunis, 2001; Shivdasani and Ingham, 2003; Kawase et al., 2004; Schulz et al., 2004). It is now clear that Jak/STAT signaling in GSCs does not regulate their self-renewal, but is required for their adherence to hub cells (Leatherman and Dinardo, 2010). BMP signaling remains as the primary self-renewal-promoting pathway for GSCs. The BMP ligands in the testis niche are *gbb* and *dpp*, and both are reportedly expressed in the hub and CySCs, consistent with the ability of CySCs to promote GSC self-renewal (Shivdasani and Ingham, 2003; Kawase et al., 2004). It is unclear why the GSCs might require BMPs from two cellular sources. It could be that BMP ligands only reach high enough levels for GSC self-renewal when produced from both the hub and CySCs, or there could be a difference in the ligand composition in the hub versus the CySCs (homodimers versus heterodimers, for example). Alternatively, there may be additional self-renewal factors that have not yet been identified, and these could be differentially expressed between the hub and the CySCs.

## THE HAIR FOLLICLE NICHE

Hair follicles are complex structures that cycle through periods of hair growth (anagen), followed by follicle regression (catagen). Between each growth cycle is a period of dormancy called telogen. Numerous distinct stem cell subpopulations have been identified within the hair follicle, with differing abilities to contribute to structures including the hair follicle itself, the interfollicular epidermis, and the sebaceous gland (Goldstein and Horsley, 2012). We will focus here on the stem cells supporting hair formation, called hair follicle stem cells (HFSCs, also called keratinocyte or epithelial stem cells). These stem cells are located in the upper, permanent region of the hair follicle called the bulge, and are identified by their slow-cycling behavior, and by the expression of Keratin-15 and CD34 (Cotsarelis et al., 1990; Lyle et al., 1998; Trempus et al., 2003). A second cell population, Lgr5-positive cells of the secondary hair germ (sHG), reside slightly basal to the bulge during telogen, but are also long-lived stem cells that support hair formation (Jaks et al., 2008; **Figure 1B**).

During anagen, the dividing hair matrix cells – the descendents of HFSCs – cluster around the dermal papilla and divide to produce cells that differentiate into the main structure of the hair fiber. Interspersed with the matrix cells are melanocytes, which produce melanin granules that are transferred to the matrix cells, thus pigmenting the hair shaft (Slominski et al., 2005). The stem cells supporting the production of new melanocytes each hair follicle cycle are the neural crest-derived melanocyte stem cells (MlSCs; note: melanocyte and mesenchymal stem cells (MsSCs) are both commonly abbreviated as “MSCs”; since we discuss both here, we will use different abbreviations). When MlSCs are not maintained, hair graying results (Nishimura et al., 2005). The MlSCs also reside in the bulge and sHG, sharing a niche with the HFSCs (Nishimura et al., 2002). Just as in the *Drosophila* testis niche, the HFSCs and MlSCs must coordinate their mitotic behavior, so that their differentiated cell types accumulate at the same time to accomplish hair pigmentation during anagen.

Several recent reports suggest that MlSCs rely on HFSCs for signals that regulate their behavior at multiple stages of the hair follicle cycle. In early anagen, Wnt signaling in HFSCs promotes proliferation and differentiation of stem cells into matrix cells (Van Mater et al., 2003; Lowry et al., 2005). Recent work indicates that MlSCs also respond to Wnt signaling: loss of β-catenin specifically in MlSCs led to a strong reduction in the number of differentiated melanocytes, and a reduction in the MlSC mitotic rate (Rabbani et al., 2011). Thus, both stem cell populations are activated during anagen by Wnt signaling. While the MlSCs do not express Wnt ligands, several Wnts are specifically upregulated in the bulge and sHG HFSCs in early anagen (Rabbani et al., 2011). In addition, *wntless*, which is required for Wnt ligand secretion, is also strongly expressed in the HFSCs during early anagen (Fu et al., 2009). Thus, while this work could not exclude an additional contribution of Wnt ligands from other sources, it is clear that one source of Wnts are the HFSCs (Rabbani et al., 2011). Thus the HFSCs promote entry of the MlSCs into anagen.

Rabbani et al. also tested the effect of constitutive HFSC Wnt signaling on neighboring MlSCs. They found that HFSC β-catenin stabilization had a strong non-autonomous effect on MlSCs, dramatically increasing their proliferation rate (Rabbani et al., 2011). In these dramatically expanded bulges, the gene encoding the secreted factor *endothelin1* was strongly upregulated*. Endothelin1* is a known transcriptional target of Wnt signaling in colon cancer cells, and is also a mitogen for melanocytes (Kim et al., 2005; Saldana-Caboverde and Kos, 2010). The authors went on to demonstrate that MlSCs express endothelin receptors, and injection of a pharmacological endothelin inhibitor prevented the MlSC expansion observed with constitutive Wnt in HFSCs, indicating that the MlSC expansion was indeed mediated via HFSC-secreted endothelin (Rabbani et al., 2011). Thus, HFSCs support entry of MlSCs into anagen via expression of both Wnts and Endothelin1.

Another recent report has implicated *endothelin2* in coordination of the HFSCs and MlSCs. Chang et al. identified an unusual phenotype – when HFSCs were mutant for the transcription factor *Nfib,* the two stem cell populations lost their coordinated behavior, and MlSCs differentiated into melanocytes during telogen, when they should have remained as quiescent stem cells. The authors found that *endothelin2* was overexpressed in *Nfib*-mutant hair follicles, and that NFIB binds directly to regulatory elements near the *endothelin2* gene. Unlike *endothelin1, endothelin2* levels were not affected by Wnt signaling. Thus, *Nfib* expression in HFSCs is required to repress *endothelin2* expression, thus promoting quiescence of neighboring MlSCs during telogen (Chang et al., 2013).

MlSCs stop dividing and become quiescent in mid-anagen of each hair follicle cycle, and Nishimura et al. have identified TGFβ as a key factor that promotes their mitotic quiescence at this stage Cultured MlSCs responded to TGFβ by withdrawing from the cell cycle, and loss of the TGFβ receptor II in MlSCs led to progressive hair graying in mice. These mice had ectopically differentiated melanocytes present in the bulge, presumably from a failure to establish quiescence, and thereby maintain a long-term bulge stem cell population (Nishimura et al., 2010). While the TGFβ ligand was detected in the niche, the *source* of the ligand was initially unclear. A more recent report has clarified that the HFSCs are the source of this ligand, as production of TGFβ ligand requires HFSC expression of collagen XVII (Tanimura et al., 2011). Thus, HFSCs also regulate entry of MlSCs into quiescence in mid-anagen via expression of TGFβ.

## THE BONE MARROW NICHE

Hematopoietic stem cells (HSCs) are some of the best studied of all adult stem cells, but their* in vivo* niche in the bone marrow is exceedingly complex. Initial reports implicated the region next to the bone surface, called the endosteum, as the niche (Nilsson et al., 2001; Calvi et al., 2003; Zhang et al., 2003; **Figure 1C**). Transplanted HSCs homed to the endosteum, and osteoblast lineage expansion, either via parathormone treatment or BMP receptor 1A inhibition, caused excess HSCs to accumulate. However, subsequent manipulations of osteoblasts produced mixed results – some reports found that osteoblast loss led to HSC loss (Visnjic et al., 2004), while other reports found no effect, or even *loss* of HSCs upon osteoblast expansion (Lymperi et al., 2008; Ma et al., 2009; Schepers et al., 2012). Subsequent reports suggested that osteoblasts *per se* were not so important, but rather, some population of osteoprogenitor cells may be the critical niche component for HSCs (Chitteti et al., 2010; Nakamura et al., 2010; Calvi et al., 2012).

In 2005, Kiel et al. (2005) identified a new method to distinguish HSCs from early non-stem progenitor cells using the SLAM family of cell surface receptors. Surprisingly, they reported that many HSCs localize next to the endothelial cells that make up the blood vessels, or sinusoids, in the bone marrow, and only a few HSCs are found on the endosteum. Thus, the authors proposed the existence of two distinct niches for HSCs. It remains an open question whether the endosteal and vascular niches both carry out a similar function, or whether they have unique roles to play. For example, some have argued that one niche is where the more primitive, quiescent HSCs reside, while the other niche harbors activated HSCs or early differentiating progenitor cells (Ehninger and Trumpp, 2011; Ding and Morrison, 2013).

The bone marrow is also the home of a second stem cell population, the MsSCs, also known as skeletal or stromal stem cells. These stem cells produce cells which differentiate into bone, cartilage, and fat, and they have been classically defined by their ability to regenerate a hematopoietic environment in an ectopic *in vivo* location (Tavassoli and Crosby, 1968). MsSCs with this regenerative capacity have recently been molecularly identified as MCAM+, CD146+ cells, and these cells reside next to the sinusoids in the bone marrow (Sacchetti et al., 2007; **Figure 1C**). Another group identified nestinGFP-positive cells as MsSCs, based on their ability to form clonal self-renewing mesenchymal spheres (“mesenspheres”), and differentiate into mesenchymal lineages *in vitro* and *in vivo* (Mendez-Ferrer et al., 2010). NestinGFP-positive cells also reside next to blood vessels, and interestingly, nearly all HSCs were found in the immediate vicinity of a nestinGFP-positive cell (even the HSCs at the endosteum were near a perivascular nestinGFP-positive cell).

Several experiments have provided strong evidence that MsSCs are a key component of the HSC niche. First, nestinGFP-positive MsSCs express high levels of genes required for HSC maintenance, including* Cxcl12, SCF, interleukin-7, angiopoietin-1, Vcam1,* and *osteopontin* (Mendez-Ferrer et al., 2010)*.* Ablation of nestinGFP-positive cells resulted in a rapid reduction in the numbers of HSCs in the bone marrow, accompanied by mobilization of HSCs to the spleen. Lethal irradiation of mice depleted for nestinGFP-positive cells showed markedly reduced homing of hematopoietic progenitors to the bone marrow, suggesting that their niche is compromised. HSC mobilization is known to be under circadian control via signals from the sympathetic nervous system, and nestinGFP-positive cells are in fact innervated with sympathetic neurons from the blood vessels (Mendez-Ferrer et al., 2008, 2010). HSC mobilization can be induced by granulocyte colony-stimulating factor (G-CSF), which leads to down regulation of HSC self-renewal factors (Semerad et al., 2005; Katayama et al., 2006). Mendez-Ferrer et al. found that G-CSF caused down-regulation of HSC self-renewal factors specifically in nestinGFP-positive cells, as well as a decrease in the nestinGFP-positive cell proliferation levels (Mendez-Ferrer et al., 2010). Parathormone treatment, which was previously shown to expand HSC numbers (presumably by increasing the size of the niche), led to a doubling in the number of nestinGFP-positive cells, while activation of parathormone signaling in only differentiated osteoblasts had no effect on HSC numbers (Mendez-Ferrer et al., 2010; Calvi et al., 2012). Finally, cotransplantation of MsSCs along with HSCs during transplantation greatly improved HSC engraftment and self-renewal (Masuda et al., 2009; Ahn et al., 2010). All these data support the notion that MsSCs help support the self-renewal of HSCs.

Significant questions remain about the exact identity of the perivascular MsSCs that support HSC self-renewal. Confusingly, it seems that nestinGFP-positive cells do not actually express the *nestin* gene or *nestin-*CRE (Ding et al., 2012). Ding et al. identified leptin receptor-expressing perivascular cells as being necessary sources of the HSC self-renewal factors SCF and CXCL12 (Ding et al., 2012; Ding and Morrison, 2013). While the described nestinGFP-positive MsSCs and the leptin receptor-positive cell populations likely have significant overlap, it is unclear whether they represent identical cell populations. Ding et al. also found that tissue-specific knock-out of *Scf* or *Cxcl12* from endothelial cells caused HSC depletion, suggesting that this cell population is also required for HSC self-renewal (Ding et al., 2012). In addition to the above-mentioned niche components, macrophages and osteoclasts have also been implicated in HSC renewal (Kollet et al., 2006; Winkler et al., 2010; Chow et al., 2011; Mansour et al., 2012; **Figure 1C**). Thus, MsSCs are one of several niche components required for HSC self-renewal.

In summary, three niches have now been found to use stem cells to support other stem cells, representing a new paradigm in niche biology. In two of the niches, the differentiating progeny function together, necessitating coordination of the stem cell populations. In mammals, MsSCs are found in many tissues, not just the bone marrow, and these cells could be involved in maintaining other stem cell populations in addition to HSCs. MsSCs are frequently used in therapeutic stem cell treatments, due to their safety and the ease with which they can be obtained (Lee, 2012). Future work will determine whether their therapeutic effects may be mediated primarily via endogenous stem cell populations, or whether they act on all cells equally. MsSCs also appear to support another type of undifferentiated cell – cancer cells. MsSCs are recruited to, and envelope tumors, and have been implicated as a niche for breast and leukemia cancer stem cells (Liu et al., 2011; Doan and Chute, 2012; Droujinine et al., 2013). In the future, as we learn more about the normal signaling between stem cell populations in niches, our ability to manipulate these cells *in vivo* will improve, increasing our capacity to understand and treat disease states.

## Conflict of Interest Statement

The author declares that the research was conducted in the absence of any commercial or financial relationships that could be construed as a potential conflict of interest.
